# Utility of CMV-Specific Immune Monitoring for the Management of CMV in Solid Organ Transplant Recipients: A Clinical Update

**DOI:** 10.3390/diagnostics11050875

**Published:** 2021-05-13

**Authors:** Katya Prakash, Aditya Chandorkar, Kapil K. Saharia

**Affiliations:** 1 Division of Infectious Diseases, University of Maryland School of Medicine, Baltimore, MD 21201, USA; kprakash@ihv.umaryland.edu; 2 Division of Infectious Diseases and International Medicine, University of Minnesota, Minneapolis, MN 55455, USA; chand716@umn.edu

**Keywords:** CMV, solid organ transplant, CD4+ T cell, CD8+ T cell, cell-mediated immunity, ELISpot, flow cytometry, ELISA

## Abstract

Cytomegalovirus (CMV) is one of the most important opportunistic infections in solid organ transplant (SOT) recipients. However, current techniques used to predict risk for CMV infection fall short. CMV-specific cell mediated immunity (CMI) plays an important role in protecting against CMV infection. There is evidence that assays measuring CMV-CMI might better identify SOT recipients at risk of complications from CMV compared to anti-CMV IgG, which is our current standard of care. Here, we review recently published studies that utilize CMV-CMI, at various points before and after transplantation, to help predict risk and guide the management of CMV infection following organ transplantation. The evidence supports the use of these novel assays to help identify SOT recipients at increased risk and highlights the need for larger prospective trials evaluating these modalities in this high-risk population.

## 1. Introduction: CMV and Its Burden on SOT

Human cytomegalovirus (CMV) infection is associated with considerable morbidity and mortality in the solid organ transplant (SOT) population [1,2]. It is the most common viral infection among SOT recipients, with an incidence of nearly 20% within the first 12 months post-transplant, despite use of anti-viral prophylaxis [3]. In SOT recipients, CMV infection can occur as a primary infection, donor-derived infection, or because of reactivation of latent virus in the setting of immunosuppression.

CMV can be characterized as (1) infection, which is defined as evidence of replicating virus in blood or tissue, irrespective of symptoms, or (2) disease, which is defined as CMV infection with accompanying symptoms. CMV disease can be further categorized as (a) CMV syndrome, in which individuals may develop fevers, malaise, and/or bone marrow suppression or (b) tissue invasive disease [4,5]. In addition to these direct effects of CMV infection, CMV is associated with significant indirect effects that can impact long-term patient and graft survival. The immunomodulatory effects of CMV may increase the incidence of bacteremia [6], increase risk of other opportunistic infections [7,8,9], lead to allograft rejection [10,11,12] or graft failure [11], and predispose SOT recipients to post-transplant lymphoproliferative disease (PTLD) [13].

The risk of CMV infection following organ transplantation is increased in recipients receiving lymphodepleting immunosuppression such as anti-thymocyte globulin (rATG) or alemtuzumab [14,15], seronegative recipients of organs from CMV seropositive donors (D+/R−) [16], and in lung [17] and small intestinal [18] transplant recipients. Allograft rejection and its treatment also increase the risk for CMV infection [19].

There are two strategies for managing CMV following organ transplantation: (1) universal anti-viral prophylaxis and (2) pre-emptive therapy, which involves routine monitoring of CMV viral load with prompt initiation of therapy once a pre-established viral load threshold is met. Each has its own distinct advantages and disadvantages. For high-risk patients, anti-viral prophylaxis reduces the incidence of early CMV disease resulting in improved patient mortality and graft survival. However, anti-viral prophylaxis is associated with higher rates of late-onset CMV disease, increased drug costs, and drug toxicities such as leukopenia [4,20,21]. Conversely, pre-emptive therapy is associated with lower rates of late-onset CMV disease and reduced drug toxicities, but it results in higher laboratory and surveillance costs and potential failure to prevent indirect sequelae of infection [22,23]. While there are several clinical trials comparing these two strategies in kidney [21,24,25,26] and liver transplant recipients [27,28], there is a dearth of data for thoracic transplant recipients.

Predicting CMV disease in organ transplant recipients is difficult. While quantitative PCR is routinely used to monitor viral DNAemia, active CMV disease does not always correlate with the presence of DNAemia, and a proportion of patients may have detectable viral loads without development of clinical disease. There is growing interest within the transplant community to utilize assays that measure CMV-specific T cell immunity to help stratify an individual’s risk for CMV disease following transplantation. These assays assess cytokine release, specifically interferon-gamma (IFN-γ), from cells following stimulation with CMV-specific antigens. Several clinical uses for these assays have been proposed:

1. Assessment of CMV T cell immunity prior to transplantation to predict risk of CMV infection post-transplantation;
2. Assessment of CMV T cell immunity during or at the end of prophylaxis to predict risk of CMV infection;
3. Assessment of CMV T cell immunity upon completing treatment of CMV infection to determine the need for secondary prophylaxis or predict risk of CMV relapse.

In this review, we will briefly discuss the host immune response to CMV infection, describe the current assays in use to assess CMV-specific cell-mediated immune response (CMI), and review the clinical evidence supporting the use of these assays in the solid organ transplant population, with a specific focus on clinical studies published within the past five years.

## 2. Host Immune Response to CMV

During natural human CMV infection, CMV elicits strong innate, humoral, and cellular immune responses. Following infection, CMV is first detected by the innate immune system via pathogen recognition receptors that detect glycoprotein B (gB) on the envelope of CMV. This in turn triggers the production of type I interferons (IFNs) and inflammatory cytokines such as tumor necrosis factor-α (TNF-α) and interleukin-6 (IL-6) [29]. Activation of innate immune responses initiates the production of antibodies, which begin to emerge within two to four weeks of primary CMV infection [30]. The major target for neutralizing antibodies to CMV is gB, which is integral to cellular attachment and penetration [31]. While the generation of antibodies is important in reducing viral dissemination and limiting the severity of disease [32], the presence of CMV antibodies alone does not confer protection [33,34]. In SOT recipients, a detectable anti-CMV IgG does not prevent CMV disease following organ transplantation [35,36]. In fact, among CMV-seropositive transplant recipients (R+), transplanting organs from CMV-seropositive donors (D+) increased the risk of CMV infection two-fold compared to transplanting organs from CMV-seronegative donors (D−) [37]. Additionally, seroconversion has not been found to be a useful predictor of future CMV disease in D+/R− patients [38].

The sustained control of CMV infection is predominantly mediated by the cell-mediated immune response. The importance of cell-mediated immunity in the control of CMV viral replication was first recognized in murine CMV studies in which the selective depletion of CD4+ and CD8+ T lymphocytes increased susceptibility to CMV infection [39,40] and the adoptive transfer of viral-specific T lymphocytes conferred protection against murine CMV [41].

In humans, numerous studies highlight the essential role both CD8+ and CD4+ T cells play in controlling and protecting against CMV infection. The development of CMV-specific CD8+ T cell responses following bone marrow transplantation (BMT) correlate with protection and recovery from CMV disease [42]. Pivotal studies by Riddell et al. and Walter et al. demonstrated that the infusion of CMV specific CD8+ T cells restored CMV-specific immunity in allogeneic BMT recipients [43,44]. In renal transplant recipients, the presence of dominant CD8+ T cell responses limited viremia and protected against disease [45], and high frequencies of CMV-specific CD8+ T cells were shown to correlate with protection against CMV disease in heart and lung transplant recipients [46].

While CMV-specific CD8+ T cell responses dominate early responses to primary CMV infection and are responsible for containment of virus-infected cells [47], there is mounting evidence that CMV-specific CD4+ T cell responses are pivotal for protection against CMV infection [48,49]. Sester et al. demonstrated that in the first few months following organ transplantation, a decrease in CMV-specific CD4+ T cell frequency preceded clinical symptoms of CMV disease [50]. Additionally, lower CMV-specific CD4+ T cell responses were associated with CMV replication in seropositive kidney [51] and liver transplant recipients [52]. In a longitudinal study of renal transplant recipients, CMV-specific effector memory CD4+ T cell responses were delayed in individuals with symptomatic CMV disease in contrast to those with asymptomatic infection [53]. Finally, adoptive transfer CMV-specific CD4+ T cells dramatically reduced CMV viral load in allogeneic stem cell transplant recipients [54]. Although the role of CMV-specific CD4+ T cells was considered to be primarily indirect, through the maintenance of virus-specific antibody responses and expansion of CD8+ T cell populations, studies now suggest that CMV-specific CD4+ T cells may also play a direct role in killing of CMV-infected cells [55].

## 3. CMV-Specific T Cell Assays

There are several assays capable of measuring CMV-specific cellular-mediated immune responses (CMI). These assays include enzyme-linked immunosorbent assay (ELISA), enzyme-linked immunosorbent spot assay (ELISpot), and intracellular cytokine staining (ICS) with flow cytometry. In general, they all rely on the detection of IFN-γ following stimulation of whole blood or peripheral blood mononuclear cells (PBMCs) with CMV-specific antigens or peptides. ICS and flow cytometry may also measure other cytokines such as interleukin-2 (IL-2) and TNF-α. For quality control, these immune assays include a positive control and negative control to monitor for errors in laboratory processing. The positive control, which uses superantigens such as phytohemagglutinin or staphylococcal enterotoxin B to stimulate T cells, identifies individuals with T cells that may be anergic due to effects of immunosuppression or low lymphocyte counts. The negative control, which can include media alone, neutral buffer, or “mock” antigen, determines non-specific background reactivity (see Table 1).

### 3.1. Intracellular Cytokine Staining (ICS) and Flow Cytometry

The use of ICS and flow cytometry provides an in-depth analysis of CMV-specific cellular immunity through the concurrent detection of cell surface markers and cytokines. To perform ICS, either whole blood or PBMCs are first incubated with CMV peptides or whole virus lysate, with or without a co-stimulatory molecule (i.e., CD28/49d antibody), for 6–18 h to stimulate the production of various cytokines. T cells are then fixed and stained using fluorescent antibodies against cell surface markers and cytokines of interest, allowing one to determine not just the frequency of cytokine-producing T cells but also the source of cytokine production (CD4+ or CD8+ T-cells). The widespread adoption of ICS/flow cytometry to measure CMV-CMI has been limited primarily by its high cost and because the assay is labor-intensive. Additionally, there is a lack of standardization, as no consensus exists on the appropriate cutoff (frequency of cytokine-positive T cell) for defining protection from CMV. Recently, Eurofins Viracor, Inc. introduced the CMV T Cell Immunity Panel (TCIP), which utilizes ICS/flow cytometry to assess CMV CMI. It is the only commercially available assay measuring CMV-CMI in the United States. CMV-specific CD4+ or CD8+ responses >0.2% are considered indicative of CMV-CMI.

### 3.2. Quantiferon-CMV (QFN-CMV)

The QuantiFERON-CMV (Qiagen) measures CD8+ T cell IFN-γ responses to a variety of HLA class I restricted CMV T cell epitopes. In this assay, whole blood is incubated with 21 CMV peptide epitopes including pp65, IE-1, IE-2, pp50, and gB, for 18–24 h, after which the supernatants are harvested, and the levels of IFN-γ are measured using a standard enzyme linked immunosorbent assay (ELISA) [56,57]. The manufacturer’s recommended cutoff for reactivity is 0.2 IU/mL. However, several papers have suggested that 0.1 IU/mL may be a more appropriate cutoff for immunosuppressed patients [58,59]. QFN-CMV is available for commercial use in Europe (CE marked) but is limited to research use only in the United States.

One heavily debated limitation of the QFN-CMV is that its use of HLA-restricted CMV epitopes may limit some individuals’ ability to recognize and respond to the antigenic determinants [60]. Studies in SOT recipients have found that up to 30% of R+ individuals lack CMV-CMI pre-transplantation when using the QFN-CMV assay [61,62]. However, a recent study evaluating CMV-CMI using a lymphoproliferation assay in CMV-seropositive healthy individuals with a negative QFN-CMV result found that individuals with discordant results exhibited lower CD4+ and CD8+ T cell proliferation in response to stimulation with CMV lysate and exhibited lower quantitative levels of anti-CMV IgG [63]. These data suggest that a negative QFN-CMV in CMV-seropositive individuals may not be related to lack of response to HLA-restricted epitopes but rather identifies a subgroup of individuals that truly have low humoral and cellular responses as has been described in patients receiving hemodialysis and in patients with cirrhosis [64,65,66].

### 3.3. CMV-ELISpot

The enzyme-linked immunosorbent spot (ELISpot) assay measures IFN-γ produced by both CD4+ and CD8+ cells in response to CMV antigen stimulation. The production of IFN-γ is quantified by measuring the number of spot forming units (sfu) in a given number of PBMCs. There are currently two commercially available assays: T-Track CMV (Lophius Biosciences GmbH, Regensburg, Germany) and T-SPOT.CMV (Oxford Immunotec Ltd., Abingdon, UK). Both assays are available commercially in Europe (CE marked). CMV ELISpot assays are performed using PBMCs isolated from whole blood. PMBCs are placed in wells and stimulated with CMV antigens, IE-1 and pp65, either as urea formulated proteins (T-Track CMV) or peptides (T-SPOT.CMV). Following 17–21 h of incubation, the secreted IFN-γ is bound by specific antibodies within the wells, and a second, enzyme-linked antibody is utilized to detect antibody-bound IFN-γ by producing insoluble precipitates or spots [67]. Although CMV-ELISpot assays are highly sensitive and commercial assays allow for standardization, there is no defined cutoff for test positivity. Studies utilizing ELISpot have used different cutoffs for positivity with ranges between 5 and 50 sfu per 200,000 PBMC [4]. Additionally, while CMV-ELISpot assays can detect both CD4+ and CD8+ T cell responses, they cannot distinguish between these responses.

## 4. Factors Impacting CMV-Specific T Cell Mediated Immunity

Before reviewing the clinical utility of CMV T cell assays, it is important to first review factors that may influence CMV-CMI in the setting of organ transplantation.

Following organ transplantation, CMV-specific T cell responses decline over the first couple months post-transplantation, with gradual increases in CMV-specific T cell responses over subsequent months [68]. However, the kinetics of CMV-specific CD4+ responses differ from those of CD8+ T cells. Sester et al. demonstrated that CMV-specific CD4+ T cell frequencies were significantly lower two months after transplantation when compared to pre-transplant levels and gradually increased to pre-transplant levels by 12 months post-transplantation. In contrast, CMV-specific CD8+ T cell responses declined rapidly within the first two weeks after transplantation but rebounded to pre-transplant levels by 60 days post-transplantation [50].

Frequencies of CMV-specific T cells following transplantation may also be influenced by induction immunosuppression. Jarque et al. demonstrated that lymphocyte depleting antibodies (rATG) caused greater reductions in the frequency of CMV-specific T cell responses than anti-IL2 receptor antibodies (basiliximab, anti-IL2RA) [69]. These results were recently confirmed by Kumar et al. [68]; however, data published by Abate et al. failed to find any impact of rATG on CMV-specific immune responses [70]. Maintenance immunosuppression, specifically calcineurin inhibitors, has also been shown to exhibit direct suppressive effects on CMV-specific T cell reactivity [71,72].

Finally, the impact of anti-viral prophylaxis on the development of CMV-CMI has been a subject of debate. A study comparing early anti-viral prophylaxis (within three days of transplantation) to delayed anti-viral prophylaxis (initiated on day 14 after transplantation) in D+/R− SOT recipients failed to find any differences in CMV-CMI between the groups [72]. Additionally, anti-viral prophylaxis administered for 100 days or 200 days did not influence CMV-CMI. Despite use of anti-viral prophylaxis, up to 20% of individuals developed CMV-CMI by day 90 post-transplantation [72]. However, a recent randomized control trial comparing anti-viral prophylaxis to pre-emptive monitoring in D+/R− liver transplant recipients found significantly higher CMV-specific T cell responses in the group randomized to pre-emptive monitoring [28]. Stronger CMV-CMI as measured by QFN-CMV was also noted in SOT D+/R− recipients who received pre-emptive therapy compared to anti-viral prophylaxis [73].

## 5. Clinical Utility of CMV Cell-Mediated Immunity (CMI) Assays

Over the past two decades, there have been a number of studies published evaluating the clinical utility of CMV-CMI assays. These studies have consistently demonstrated that high CMV-CMI predicts protection against CMV outcomes whereas low CMV-CMI increases the likelihood of CMV outcomes. Despite these consistent findings, CMV-CMI assays are not routinely integrated into clinical practice. In the United States, CMV-CMI assays are available only for research or as laboratory-developed tests with the exception of the Eurofins Viracor TCIP. The main reasons for this are (1) poorly defined thresholds for positive and negative results, (2) variability in CMV antigen stimulation protocols (use of whole cell lysate vs. peptide pools, use of IE-1 vs. pp65), and (3) heterogeneity in the populations being studied (type of organ transplant, donor–recipient serostatus, use of antiviral prophylaxis vs. pre-emptive monitoring). Additionally, most data supporting CMV-CMI use are observational, with only a few interventional studies where treatment decisions were made based on the results of a given CMV-CMI assay. In this section, we will review the existing clinical data assessing CMV-CMI assays both pre- and post-transplantation to predict the risk of CMV infection, focusing primarily on data accrued over the past five years (see Table 2).

### 5.1. Can Pre-Transplant CMV CMI Predict Risk of CMV Disease Post-Transplant?

At present, donor and recipient CMV IgG is the only test utilized to stratify the risk of CMV infection following organ transplantation. While D+/R- patients are at highest risk of CMV disease, not all D+/R− recipients develop CMV infection in the absence of anti-viral prophylaxis. Moreover, late onset CMV develops in 8–34% of R+ recipients, depending on the type of organ transplanted [89,90], highlighting that risk of CMV post-transplantation is more granular than just CMV serostatus. There is a growing body of evidence that CMV-CMI assays performed prior to transplantation might provide a better assessment of CMV risk in both high (D+/R−) and intermediate risk (R+) recipients.

One of the first studies to assess whether pre-transplant CMV-CMI could predict post-transplant CMV events was a single center retrospective study using a CMV ELISpot assay in D+/R− and R+ kidney transplant recipients [91]. Investigators found that 43% (12/28) of D+/R− patients had detectable pre-transplant CMV-CMI, although the magnitude of the CMV-specific response was lower among seronegative recipients when compared to seropositive recipients. Patients with post-transplant CMV infection had significantly lower pre-transplant CMV-CMI when compared to patients without CMV events following transplantation [91].

Schachtner and colleagues prospectively evaluated pre-transplant CMV-CMI in both D+/R− and R+ kidney transplant recipients as a predictor of post-transplant CMV infection using CMV ELISpot [82]. Approximately 28% of CMV seronegative recipients had evidence of CMV-CMI pre-transplantation. While pre-transplant CMV-CMI did not correlate with subsequent development of CMV infection in D+/R− recipients, D+/R− individuals with pre-transplant CMV-CMI did have significantly lower CMV viral loads at time of diagnosis and a trend toward lower incidence of CMV disease (*p* = 0.067). Among R+ individuals, pre-transplant CMV-CMI was associated with lower CMV viral loads at diagnosis, lower incidence of CMV disease, and reduced need for anti-viral therapy when compared to R+ individuals without pre-transplant CMV-CMI.

A number of additional observational studies have similarly demonstrated a higher risk of CMV events among SOT recipients without CMV CMI pre-transplantation using CMV ELISpot [81,90], QFN-CMV [58], and ICS/flow cytometry [81,82], although some differences in predictive ability of CMV-specific CD4+ versus CD8+ responses were noted based on induction immunosuppression [81,82]. Recently, a large multicenter, prospective, observational study of 243 renal transplant recipients found lower median sfu to both IE-1 and pp65 among individuals that developed CMV events post-transplant compared to those without a CMV event. The negative predictive value (NPV) using a threshold of >40 sfu for IE-1 or pp65 against post-transplant CMV events was >95% [68].

In the only prospective, interventional study published, Jarque et al. [69] evaluated pre-transplant CMV CMI using T-SPOT.CMV (Oxford Immunotec, Ltd.) to predict CMV infection following kidney transplantation. Investigators stratified 160 kidney transplant recipients (D+/R+) by baseline responses to IE-1 and then randomized individuals to either pre-emptive monitoring or 3 months of anti-viral prophylaxis. Individuals without pre-transplant CMV-CMI randomized to pre-emptive monitoring demonstrated a significantly higher incidence of CMV infection than individuals with pre-transplant CMV-CMI (73% vs. 44.4%, OR 3.44, 95% CI 1.3–9.08). Additionally, the incidences of CMV infection requiring treatment (53.3% vs. 18.5%, OR 5.03, 95% CI 1.86–13.57) and CMV disease (20% vs. 3.7%, OR 6.5, 95% CI 1.22–34.59) were significantly higher among those without versus with pre-transplant CMV-CMI.

Data accrued over the past several years consistently show that individuals with pre-transplant CMV-CMI are at lower risk of developing CMV infection following transplantation. For those with pre-transplant CMV-CMI that do develop CMV infection, infection tends to be milder (lower CMV viral load, less invasive disease). Induction immunosuppression could impact the predictive value of CMV-CMI pre-transplantation; however, this has not been consistently demonstrated. Finally, much of the data come from kidney transplant recipients. A recent study in liver transplant recipients following a pre-emptive CMV monitoring strategy failed to find a correlation between pre-transplant CMV-CMI and post-transplant CMV viremia. Although, when restricting the analysis to episodes of CMV viremia occurring two months after liver transplantation, the presence of CMV-CMI pre-transplantation did appear to confer protection against CMV viremia [92]. Whether the predictive value of pre-transplant CMV-CMI as reported in kidney transplant recipients applies to other organ transplant recipients is not known. Well-designed studies addressing the predictive ability of pre-transplant CMV-CMI in thoracic transplant and liver transplant recipients are needed.

### 5.2. Can Assessment of CMV-CMI at the End of Anti-Viral Prophylaxis or Early after Transplantation Predict Risk of Subsequent CMV Disease?

While anti-viral prophylaxis is highly effective at decreasing incidence of CMV DNAemia and disease, it leads to higher costs, toxicities and increases rates of late-onset CMV infection. Measuring CMV-CMI at the end of prophylaxis (EOP) may allow for individualization of prophylaxis by identifying those who would benefit from early cessation of prophylaxis versus those requiring extended prophylaxis and/or closer monitoring following discontinuation of anti-viral prophylaxis.

The largest study to date by Kumar et al. [68] measured CMV-CMI in a cohort of kidney transplant recipients using T-SPOT.CMV. End of prophylaxis assessments for CMV-CMI were available for 368 recipients of whom 45% were D+/R−. Using a threshold of >40 sfu, the NPV of the test was 97% for pp65 (*p* < 0.0001) and 97.7% for IE-1 (*p* < 0.0001). There was a significant difference in risk of CMV event for patients with IE-1 and pp65 sfu values above the established threshold when compared to those below (3% vs. 19.5% for EOP pp65, *p* < 0.0001; 2.3 vs. 15% for EOP IE-1, *p* = 0.0006).

Another notable study by Jarque et al. [84] prospectively assessed CMV CMI at EOP to predict late onset CMV infection in D+/R+ kidney transplant recipients receiving either anti-interleukin 2 receptor antibody (anti-IL2RA, *N* = 50) or rATG (*N* = 46). CMV-CMI was assessed using T-SPOT.CMV. Similar to Kumar and colleagues, these authors found that frequencies of CMV-specific T cells at EOP (three months post-transplantation) were significantly lower in patients that developed late-onset CMV infection. There were no differences in mean CMV-CMI at EOP when comparing individuals receiving rATG or anti-IL2RA for induction immunosuppression. The authors also assessed the risk of late-onset CMV based on stratification of CMV-CMI, defining HR (high risk) as a negative test against both IE-1 and pp65, LR (low risk) as positive tests against both antigens, and IR (intermediate risk) as a negative result against 1 of the 2 CMV antigens. The risk of late-onset CMV infection gradually increased in accordance with risk stratification groups (5.6% within LR, 18.2% within IR, and 35% within HR; log-rank test = 0.006).

Two studies evaluating the predictive value of CMV-CMI at the EOP in lung transplant recipients have recently been published. Donadeu et al. [86] retrospectively evaluated CMV-CMI at EOP (6 months post-transplantation) using T-SPOT.CMV in 60 R+ lung transplant recipients. While a negative CMV (IE-1)-specific CMI at EOP was associated with late-onset CMV DNAemia, it did not predict the same in multivariable analysis. However, the absence of CMV (IE-1)-specific CMI did predict high level CMV replication defined as CMV DNA PCR >20,000 (OR 4.3, 95% CI 1.043–18.215). Westall et al. [76] performed a single center, prospective, interventional pilot study evaluating the CMV-specific T cell response using QFT-CMV. Lung transplant recipients were administered anti-viral prophylaxis for five months post-transplantation at which time QFN-CMV was performed. Patients with CMV-CMI at EOP were monitored off anti-viral therapy while those with negative CMV-CMI were maintained on variable anti-viral prophylaxis through month 11 post-transplantation, guided by QFN-CMV measurements. The authors in this study chose the detection of CMV PCR in BAL as their primary endpoint. The authors found a lower rate of CMV infection within the lung allograft in patients receiving CMV-CMI guided anti-viral prophylaxis compared to standard of care (37% vs. 58%, *p* = 0.03). A large multicenter randomized clinical trial (CYTOCOR trial) is currently underway in Spain that aims to evaluate the role of QFN-CMV in guiding primary prophylaxis duration in R+ lung transplant patients [93].

CMV-CMI has also been evaluated in two cohorts of heart transplant recipients. In the first, investigators retrospectively evaluated CMV-CMI using QFN-CMV in 44 R+ heart transplant recipients [75]. Investigators found a higher proportion of CMV infection among patients with indeterminate QFN-CMV at EOP compared to those with positive QFN-CMV results (*p* = 0.036). In a second study, authors performed a prospective, non-randomized, interventional study involving 159 heart transplant recipients (>80% R+) at a single center in which QFN-CMV was measured at EOP (100 days post-transplantation) and patients were assigned to CMV-CMI guided prophylaxis or standard of care [77]. In the CMV-CMI guided group, anti-viral prophylaxis was continued until patients had measurable CMV-CMI by QFN-CMV, while patients in the standard of care group stopped anti-viral prophylaxis at 100 days or clinicians’ discretion. Lower rates of CMV infection were noted in the group undergoing CMV-CMI guided prophylaxis (5% vs. 19%, *p* = 0.03); however, the duration of anti-viral prophylaxis was greater in the CMV-CMI guided group (155 +/− 102 days vs. 104 +/− 48 days, *p* < 0.05), with no difference in rates of leukopenia between the two groups.

Finally, several studies have evaluated whether the detection of CMV-CMI at time points preceding EOP could predict the development of subsequent CMV infection. Lee et al. [85] found that in R+ kidney transplant recipients, detection of CMV-CMI at one month post-transplant was predictive of protection with NPV of 94.5% and 97.6% in pp65 and IE-1 assays, respectively. Jarque et al. [84], in addition to assessing the utility of CMV-CMI at EOP as noted above, evaluated whether earlier detection of CMV-CMI would have better predictive ability. They found that kidney transplant recipients receiving T-cell depleting induction therapy had profound abrogation of CMV-CMI at earlier time-points while those receiving anti-IL2RA induction had a far less pronounced reduction in CMV-CMI at earlier time-points. Thus, detection of CMV-CMI after the first month following transplantation was more accurate in predicting CMV infection risk only in patients receiving anti-IL2RA induction.

Although most of the recent published data involve assessment of CMV-CMI using QFN-CMV or CMV ELISPOT, three studies in the past several years have assessed CMV-CMI using flow cytometric analysis. Snyder et al. performed a cross-sectional analysis of CMV-CMI in R+ lung transplant recipients by stimulating PBMCs with CMV pp65 and IE-1 peptide pools. Using flow cytometry, they identified polyfunctional T cell signatures (CD107a−/IFN-y+/IL-2+/TNF-a+CD4+ T cells and CD107a−/IFN-y+/IL-2+/TNF-a+CD8+ T cells) that were predictive of protection against future CMV infection [87]. In a retrospective analysis of primarily SOT recipients, Rogers and colleagues utilized the Eurofins Viracor TCIP to determine the value of CMV-CMI in predicting clinically significant CMV events [88]. Consistent with data from other CMV immune assays, the frequency of CMV-specific CD4+ T cells was significantly lower in patients with CMV events compared to those without CMV events (median 0.13 vs. 0.73, *p* = 0.002). While the frequency of CMV-specific CD8+ T cells was also lower in those with CMV events, the results did not reach statistical significance (median 0.46 vs. 0.9, *p* = 0.08). Finally, Gabanti and colleagues used an in-house CMV T cell assay based on T cell stimulation by autologous HCMV-infected dendritic cells to determine CMV-CMI using flow cytometry [94]. In their cohort of 53 R+ kidney transplant recipients, recipients with low peak viral load CMV infection (peak VL < 3 × 10^5^ DNA copies/mL) had higher CMV-specific CD4+ and CD8+ T cell responses when compared to recipients with higher peak CMV viral load infection (peak VL >3 × 10^5^ DNA copies/mL).

Not all studies have found a clear association between CMV-CMI and protection against future CMV infection. Recently, Fernandez-Ruiz et al. reported on the assessment of CMV-CMI using QFN-CMV at EOP in R+ kidney transplant recipients receiving rATG induction [79]. Their data demonstrated suboptimal accuracy for predicting 1-year CMV infection rates (45.8% in persons with non-reactive or indeterminate results vs. 36.1% in persons with reactive results, *p* = 0.244). Modifying the manufacturer’s interpretative criteria led to some improvement in diagnostic performance. The negative results in this study could be attributed to the inability to detect CMV-specific CD4+ T cell responses, as patients in this cohort received rATG induction and CMV-CMI was assessed using QFN-CMV. In another study, Lee et al. also failed to find a correlation between CMV-CMI assessed by QFN-CMV at 1 or 3 months post-transplantation and subsequent CMV viremia, but they did find better accuracy using CMV ELISPOT [85].

In summary, assessment of CMV-CMI at the end of prophylaxis consistently demonstrates a higher risk of late-onset CMV events among individuals without CMV CMI across organ groups. Assessment of CMV-CMI at time-points as early as one-month post-transplantation can predict subsequent risk of CMV infection, but the predictive value may be impacted by induction immunosuppression. Finally, there appear to be assay-specific differences in prediction of CMV infection or disease, with several studies demonstrating poor correlation between CMV-CMI detected by QFN-CMV and subsequent risk of CMV infection.

### 5.3. Can Assessment of CMI at End of Treatment Determine Risk of Relapse?

The risk of recurrent CMV infection is estimated to be between 20% and 30% [95,96,97]. Lung transplant recipients, those with persistent CMV DNAemia despite 21 days of therapy, and CMV seronegative patients at time of primary infection are at highest risk for relapsed infection [95]. Small observational studies have proposed a role for CMV-CMI testing at the end of therapy to predict risk of relapsed CMV infection. Chiereghin et al. evaluated 24 high and intermediate risk heart transplant recipients who developed CMV infection with the QFN-CMV assay at the end of therapy (EOT) [75]. No patient with a positive CMV-CMI at EOT developed relapsed disease. In comparison, six of 15 patients (40%) with a negative or indeterminate QFN-CMV experienced a second infection. In a retrospective study by Rogers et al., six SOT recipients had CMV-CMI testing following their initial CMV event using the Viracor TCIP [88]. Five of six patients had CMV-specific CD4+ T cells >0.22% (positive response) and did not experience subsequent CMV events after discontinuation of anti-viral therapy.

Finally, the largest study to date evaluating the role of CMV-CMI in predicting the risk for CMV relapse is an interventional study conducted by Kumar et al. that enrolled all organ transplant patients with a documented episode of CMV infection [78]. CMV-CMI was assessed at EOT using the QFN-CMV assay. Anti-viral therapy was discontinued in individuals with positive QFN-CMV at EOT. In patients with a negative QFN-CMV, prophylaxis was continued for two additional months. A total of 32 patients were included. Only one of the fourteen CMV-CMI positive patients, a D+/R− lung transplant recipient, developed recurrent viremia after discontinuation of prophylaxis. In the 13 patients without detectable CMV-CMI, nine developed CMV viremia either during or after discontinuation of prophylaxis.

Although data are limited, the evidence generated to date supports the clinical assessment of CMV-CMI testing at EOT to guide decisions regarding the need for and duration of secondary prophylaxis in transplant recipients that have recovered from CMV infection post-transplantation.

## 6. Future Directions and Conclusions

The use of CMV immune monitoring to better identify SOT recipients at increased risk of CMV-related complications has long been considered an area of clinical interest and need. The development of assays measuring CMV-specific immune responses such as T-Track CMV, T-SPOT.CMV, QuantiFERON-CMV, and the Viracor TCIP have propelled this field forward. Observational studies published in the past several years evaluating the utility of these assays continue to add support for the routine clinical use of these assays. Importantly, several recent large, prospective, interventional studies have begun to provide long-awaited answers to questions about the ideal timing for performing these assays, the utility of repeated assessments of CMV-CMI, and the benefits of CMV-CMI in relation to standard of care.

However, there are still some unresolved questions about these assays including cost efficacy, impact of different immunosuppressive regimens on the predictive value of these assays, standardization of cutoff values for CMV-CMI determination, and predictive value in thoracic organ transplant recipients, as current data from interventional studies has come from kidney transplant recipients. Additionally, there are few head-to- head comparisons of the various CMV T cell immune assays. In the coming years, additional data from several ongoing prospective, interventional clinical trials evaluating CMV-CMI will be published, including one in lung transplant recipients, which hopefully will help answer some of these lingering questions.

In conclusion, there are compelling data supporting the role of CMV T cell immune assays in the management of CMV infection in SOT recipients. While we do not yet have sufficient data for the widespread adoption of these assays into routine clinical use, data accumulated over the past 5 years has brought us one step closer.

## Figures and Tables

**Table 1:** Overview of CMV T cell assays used to measure CMV-specific immune responses.

ASSAYS	BRAND NAMES	CYTOKINES MEASURED	TARGET CELLS	ANTIGEN	ADVANTAGES	DISADVANTAGES
ELISPOT	T-SPOT.CMV T-TRACK.CMV	IFN-γ	CD4/CD8	IE-1 and pp65 Peptides T-Activated CMV Proteins (IE-1, pp65)	Not Limited by HLA. Measures Both CD4/CD8 Response	Requires ELISpot Reader, No Standardization, Does Not Differentiate Between CD4 or CD8 Response
ELISA	QuantiFERON-CMV	IFN-γ	CD8	21 Epitopes Mapped Within pp65, IE-1, pp50, IE-2, gB, and pp28; HLA Class I Restricted	Standardized, not Labor-Intensive	HLA Class I-Restricted, Sensitive to Lymphopenia, Measures CD8 Response Only
FLOW CYTOMETRY	VIRACOR CMV T Cell Immunity Panel (TCIP)	IFN-γ CD69	CD4/CD8	CMV pp65 Peptide Mix or CMV Grade 2 Antigen Mix	Can Differentiate Between CD4 and CD8 Response; Potential to Measure a Variety of Cytokines and Cell Surface Markers	High Cost, No Standardization, Labor Intensive, Largely Limited to Research Use Only Except for VIRACOR TCIP

**Table 2:** Summary of the recent relevant interventional and observational studies evaluating CMV-specific cell-mediated immunity in solid organ transplant recipients.

CMV T Cell Assay	Author, Year, Location (Ref)	Organ, Donor/Recipient Status, No. Patients	Study Design and Primary Endpoint	Cutoff for Positive Test	Results	Limitations
QuantiFERON-CMV	Andreani et al., 2020, France [74]	Kidney, D+/R− *N* = 12	Observational Study of Patients with Asymptomatic Viremia, Ability of CMI to Predict Spontaneous Viral Clearance or CMV Disease	>0.1 IU/mL	Spontaneous Clearance Occurred in 6/6 (100%) Patients with CMI at Time of Viremia vs. 1/5 (20%) with no CMI, *p* = 0.02	Small Sample Size Observational Study, Single Organ
QuantiFERON-CMV	Chiereghin et al., 2018, Italy [75]	Heart, D+/R− and R+, *N* = 44	Retrospective, Utility of CMI to Predict CMV Infection in Preemptive vs. Prophylaxis Groups and to Predict Risk of Relapse After First Episode of CMV Infection	>0.2 IU/mL	In Prophylaxis Group, 66.7% with Indeterminate Result vs. 14.3% of Patients with CMI Developed CMV Infection, *p* = 0.036. No Difference in Pre-Emptive Therapy. No Patients who Developed CMI After Primary Infection Developed Relapse vs. 6/15 with Indeterminate or Negative Results, *p* = 0.032	Retrospective Study, Heterogenous Donor/Recipient Serostatus, Single Organ
QuantiFERON-CMV	Manuel et al., 2013, International [58]	All organs, D+/R−, *N* = 127)	Prospective Observational Study, Evaluation of CMI at End of Prophylaxis to Predict CMV Disease	>0.1 IU/mL	Patients with CMI at End of Prophylaxis Were Significantly Less Likely to Develop CMV Disease (6.4% vs. 22.2% vs. 58.3% for Positive vs. Negative vs. Indeterminate Result, *p* ≤ 0.001	Observational Study Only
QuantiFERON-CMV	Cantisan et al., 2013, Spain [61]	Lung, Kidney, R+ and R−, *N* = 55	Evaluating Pre-Transplant CMI to Predict Post-Transplant CMV Replication (CMV Viremia, Asymptomatic and Disease)	>0.2 IU/mL	Pretransplant CMI Can Predict Development of CMV Independent of Serostatus	Small Number, Evaluated Asymptomatic Viremia
QuantiFERON-CMV	Westall et al., 2019, Australia [76]	Lung, All, *N* = 118	Interventional, Pilot, RCT; CMI Performed at End of Prophylaxis, Patients Randomized to CMI-Guided Prophylaxis vs. Standard of Care. Primary end Point CMV Detection by PCR in BAL	>0.2 IU/mL	CMI Guided Prophylaxis Had Lower Incidence of CMV Infection in Lung Allograft Than Standard of Care	Small Pilot Study, High Number of Indeterminate and Negative QFN-CMV Test Results, Endpoint Was BAL PCR Positivity
QuantiFERON-CMV	Poglajen et al., 2020, Slovenia [77]	Heart, All, *N* = 154	Prospective, Interventional Study, Non-Randomized Evaluating CMI-Guided Prophylaxis vs. Standard of Care, Primary End Point CMV Viremia and Disease	>0.2 IU/mL	CMI Guided Prophylaxis Resulted in Lower Rates of CMV Infection (5% vs. 19%, *p* = 0.03) but Longer Duration of Anti-Viral Prophylaxis Without any Increased Rates of Leukopenia	Non-Randomized Study, Single Center
QuantiFERON-CMV	Kumar D et al., 2017, Canada [78]	All organs, All, *N* = 27	Non-Randomized Interventional Study Evaluating Utility of CMI in Guiding Secondary Prophylaxis and at Predicting Risk of CMV Relapse	>0.2 IU/mL	At End of Therapy 9/13 Patients with Negative CMI vs. 1 of 14 Patients With Positive CMI at End of Therapy Developed CMV Recurrence (*p* = 0.001)	Small Sample Size, Non-Randomized Study
QuantiFERON-CMV	Fernandez-Ruiz et al., 2020, Spain [79]	Kidney, R+ (*N* = 120)	Prospective, Observational (Non-Interventional) Study Evaluating CMI at End of Prophylaxis to Predict CMV Viremia and Disease in Patients that Received ATG Induction	>0.2 IU/mL	End of Prophylaxis CMI Did Not Predict CMV Infection	Non-Randomized Study, Non-Interventional. ATG Induction Only.
T-Track CMV	Kim et al., 2020, Korea [80]	Kidney, R+, *N* = 133	Observational Cohort Study Evaluating Pre-Transplant CMI to Predict Post-Transplant CMV Infection or Disease as Measured by CMV Antigen	>10 Spots per 200,000 Cells	Absence of Pre-Transplant CMI was Independent Risk Factor for CMV Infection, AHR 1.87	Single Center, Observational, Using CMV Antigen
T.SPOT.CMV	Jarque et al., 2020, Spain [69]	Kidney, R+, *N* = 160	Prospective, Randomized, Multicenter, Observational Study Evaluating Whether Pre-Transplant CMI Could Predict Post-Transplant CMV Infection or Disease. Patients With and Without Pre-Transplant CMI Randomized to Either Pre-Emptive or Prophylactic Strategy	20 Spots per 300,000 Cells	Patients With Negative Pre-Transplant CMI Had Higher Rates of Post-Transplant CMV Infection in Both Pre-Emptive (73.3% vs. 44.4%, OR 3.44) and Prophylaxis (33.3% vs. 4.1%, OR 11.75) Strategies.	
ELISpot	Lucia et al., 2014, Spain [81]	Kidney, R+ and R−, *N* = 129	Retrospective Case Control Study Evaluating Pre-Transplant CMI to Predict Post-Transplant CMV Infection	No a Priori Cutoff	Patients With High Pre-Transplant CMV-Specific T Cell Responses were Less Likely to Develop CMV Infection Post-Transplant	No a Priori Cutoff for ELISpot, Retrospective
ELISpot	Schachtner et al., 2017, Germany [82]	Kidney, All, *N* = 326	Prospective, Observational Trail Evaluating Pre-Transplant CMI to Predict Post-Transplant CMV Disease	No a Priori Cutoff	D+/R− and R+ With Evidence of Pre-Transplant CMI Did Not Have Lower Incidence of CMV Replication But Did Have Lower Peak CMV Viral Loads, Lower Incidence of CMV Disease, and were Less Likely to Require Anti-Viral Therapy	Single Center, Non-Interventional
T.SPOT.CMV	Kumar et al., 2019, Multicenter [68]	Kidney, All, *N* = 583	Prospective, Observational Study Evaluating Pre-Transplant CMI and End of Prophylaxis CMI to Predict CMV Infection	40 Spots per 250,000 Cells	Patients With Positive CMI Either Pre-Transplant (NPV 95%) or at End of Prophylaxis (3% vs. 19.5%, *p* < 0.001, NPV > 97%) Were Less Likely to Develop CMV Infection	
T.SPOT. CMV	Chanouzas et al., 2018, United Kingdom [83]	Kidney, All, *N* = 108	Observational Cohort to Assess CMI at End of Prophylaxis to Predict CMV Viremia	IE1 (>25 Spots/2.5 × 10^5 PBMC’s) pp65 (>50 spots/2.5 × 10^5 PBMC’s)	Individuals With CMI at End of Prophylaxis were Less Likely to Develop CMV Viremia	Small Sample Size, Different Cutoff for Positivity than Other Studies, Observational Study
T.SPOT.CMV	Jarque et al., 2018, Spain [84]	Kidney, R+, *N* = 96	Observational Study to Assess CMI at End of Prophylaxis to Predict Subsequent CMV Infection	IE1 (>25 spots/3 × 10^5 PBMC’s) pp65 (>130 spots/3 × 10^5 PBMC’s)	CMV CMI Frequencies Were Significantly Lower in Patients Developing Late-Onset CMV (*p* < 0.001 or IE-1, *p* = 0.30 for pp65)	Single Center, Observational Study
QuantiFERON-CMV CMV ELISpot	Lee et al., 2017, Korea [85]	Kidney, D+/R+ *N* = 124	Assessment of Pre- and Post-Transplant CMI Using Two IGRAs to Determine Risk of Post-Transplant CMV	pp65 30 spots/200,000 cells IE-1 10 Spots/200,000 Cells	ELISpot at One-Month Post-Transplant Predicted Risk for Late-Onset CMV Infection. No Association Noted With QFN-CMV at Any Time Point. CMV ELISpot Pre-Transplant Could not Predict Post-Transplant CMV Infection.	Small Sample Size, Observational Cohort. Most Cases of CMV were Asymptomatic and Cleared Spontaneously
T.SPOT.CMV	Donadeu et al., 2020, Spain [86]	Lung, R+ *N* = 60	Retrospective Study Evaluating CMI at End of Prophylaxis to Predict Late Onset CMV Infection and Disease	55 IE-1 Spots per 300,000 Cells	Lung Transplant Recipients With Late-Onset CMV had Significantly Lower CMI Compared to Those Who Did Not Have Late Onset CMV Infection (*p* = 0.045)	Retrospective, Small Sample Size, Non-Intervention
ICS/Flow Cytometry	Singh et al. 2020, Multicenter [28]	Liver, D+/R−, *N* = 538	RCT of D+/R− Liver Transplant Recipients Assigned to Prophylaxis vs. Pre-Emptive Therapy. As Secondary Outcome, Investigator Evaluated CMV-CMI	No Predefined Criteria	Patients Randomized to Pre-Emptive Therapy had Higher Frequency of CMV-Specific T Cells (Including Polyfunctional T Cell Responses) than Patients Randomized to Prophylaxis	Study Primarily Designed to Assess Clinical Outcome of CMV Infection Based on Prophylaxis vs. Pre-Emptive Treatment.
ICS/Flow Cytometry	Snyder et al. 2016, USA [87]	Lung, D/R status unknown, *N* = 71	Cross-Sectional Assessment of Polyfunctional CMI to Predict Protection from Subsequent CMV Infection	No Cutoff	Identified Several Polyfunctional T Cell Signatures That Predicted Protection from CMV Viremia	Small Sample Size, Single, Center, Observational Study, Samples Obtained at Variable Time Points After Transplant
ICS/Flow Cytometry	Rogers, et al. 2020, USA [88]	All Transplant, All Donor-Recipient Status, *N* = 31	Cross-Sectional Assessment of CMI in Patients with CMV Infection	0.2% CMV-Specific CD4+ T Cells 0.2% CMV-Specific CD8+ T Cells	Patients with CMV-Specific CD4 T Cell Responses were Protected from Subsequent CMV Events with PPV of Protection from CMV Event of 85%	Cross-Sectional Analysis, Small Sample Size, Heterogeneous Population

## Definitions

CMV: cytomegalovirus; SOT: solid organ transplant; CMI: cell-mediated immunity; D−: CMV seronegative donor; D+: CMV seropositive donor; R+: CMV seropositive recipient; R−: CMV seronegative recipient; D+/R−: CMV mismatch, seronegative recipient of a seropositive donor organ.

## References

[1] Limaye A.P., Bakthavatsalam R., Boeckh M., Kim H.W., Randolph S.E., Halldorson J.B., Healey P.J., Kuhr C.S., Levy A.E., Perkins J.D. (2006). Impact of Cytomegalovirus in Organ Transplant Recipients in the Era of Antiviral Prophylaxis. Transplantation.

[2] Bosch W., Heckman M.G., Diehl N.N., Shalev J.A., Pungpapong S., Hellinger W.C. (2011). Association of Cytomegalovirus Infection and Disease with Death and Graft Loss After Liver Transplant in High-Risk Recipients. Am. J. Transplant..

[3] Paya C., Humar A., Dominguez E., Washburn K., Blumberg E., Alexander B., Freeman R., Heaton N., Pescovitz M.D., Valganciclovir Solid Organ Transplant Study Group (2004). Efficacy and Safety of Valganciclovir vs. Oral Ganciclovir for Prevention of Cytomegalovirus Disease in Solid Organ Transplant Recipients. Am. J. Transplant..

[4] Kotton C.N., Kumar D., Caliendo A.M., Huprikar S., Chou S., Danziger-Isakov L., Humar A. (2018). The Third International Consensus Guidelines on the Management of Cytomegalovirus in Solid-organ Transplantation. Transplantation.

[5] Razonable R.R., Humar A. (2019). Cytomegalovirus in Solid Organ Transplant Recipients—Guidelines of the American Society of Transplantation Infectious Diseases Community of Practice. Clin. Transplant..

[6] Snydman D.R. (2006). The Case for Cytomegalovirus Prophylaxis in Solid Organ Transplantation. Rev. Med. Virol..

[7] Linares L., Sanclemente G., Cervera C., Hoyo I., Cofán F., Ricart M., Pérez-Villa F., Navasa M., Marcos M., Antón A. (2011). Influence of Cytomegalovirus Disease in Outcome of Solid Organ Transplant Patients. Transplant. Proc..

[8] Munoz-Price L.S., Slifkin M., Ruthazer R., Poutsiaka D.D., Hadley S., Freeman R., Rohrer R., Angelis M., Cooper J., Fairchild R. (2004). The Clinical Impact of Ganciclovir Prophylaxis on the Occurrence of Bacteremia in Orthotopic Liver Transplant Recipients. Clin. Infect. Dis..

[9] Duncan S.R., Paradis I.L., Yousem S.A., Similo S.L., Grgurich W.F., Williams P.A., Dauber J.H., Griffith B.P. (1992). Sequelae of Cytomegalovirus Pulmonary Infections in Lung Allograft Recipients. Am. Rev. Respir. Dis..

[10] Cerrina J., Ladurie F.L.R., Hervé P.H., Parquin F., Harari S., Chapelier A., Simoneau G., Vouhé P., Dartevelle P.H. (1992). Role of CMV Pneumonia in the Development of Obliterative Bronchiolitis in Heart-Lung and Double-Lung Transplant Recipients. Transpl Int..

[11] Hakimi Z., Aballéa S., Ferchichi S., Scharn M., Odeyemi I.A., Toumi M., Saliba F. (2017). Burden of Cytomegalovirus Disease in Solid Organ Transplant Recipients: A National Matched Cohort Study in An Inpatient Setting. Transpl. Infect. Dis..

[12] Paraskeva M., Bailey M., Levvey B.J., Griffiths A.P., Kotsimbos T.C., Williams T.P., Snell G., Westall G. (2011). Cytomegalovirus Replication Within the Lung Allograft Is Associated with Bronchiolitis Obliterans Syndrome. Am. J. Transplant..

[13] Dharnidharka V.R., Kenneth Sullivan E., Stablein D.M., Tejani A.H., Harmon W.E. (2001). Risk Factors for Posttransplant Lymphoproliferative Disorder (PTLD) in Pediatric Kidney Transplantation: A Report of the North American Pediatric Renal Transplant Cooperative Study (NAPRTCS). Transplantation.

[14] Kaminski H., Jarque M., Halfon M., Taton B., Di Ascia L., Pfirmann P., Visentin J., Garrigue I., Déchanet-Merville J., Moreau J.-F. (2019). Different Impact of rATG Induction on CMV Infection Risk in D+R− and R+ KTRs. J. Infect. Dis..

[15] Luan F.L., Samaniego M., Kommareddi M., Park J.M., Ojo A.O. (2010). Choice of Induction Regimens on the Risk of Cytomegalovirus Infection in Donor-Positive and Recipient-Negative Kidney Transplant Recipients. Transpl. Infect. Dis..

[16] Sia I.G., Patel R. (2000). New Strategies for Prevention and Therapy of Cytomegalovirus Infection and Disease in Solid-Organ Transplant Recipients. Clin. Microbiol. Rev..

[17] Zamora M.R. (2004). Cytomegalovirus and Lung Transplantation. Am. J. Transplant..

[18] Ambrose T., Sharkey L., Louis-Auguste J., Rutter C., Duncan S., English S., Gkrania-Klotsas E., Carmichael A., Woodward J., Russell N. (2016). Cytomegalovirus Infection and Rates of Antiviral Resistance Following Intestinal and Multivisceral Transplantation. Transplant. Proc..

[19] Razonable R.R., Rivero A., Rodriguez A., Wilson J., Daniels J., Jenkins G., Larson T., Hellinger W.C., Spivey J.R., Paya C.V. (2001). Allograft Rejection Predicts the Occurrence of Late-Onset Cytomegalovirus (CMV) Disease among CMV-Mismatched Solid Organ Transplant Patients Receiving Prophylaxis with Oral Ganciclovir. J. Infect. Dis..

[20] Humar A., Lebranchu Y., Vincenti F., Blumberg E.A., Punch J.D., Limaye A.P., Abramowicz D., Jardine A.G., Voulgari A.T., Ives J. (2010). The Efficacy and Safety of 200 Days Valganciclovir Cytomegalovirus Prophylaxis in High-Risk Kidney Transplant Recipients. Am. J. Transplant..

[21] Khoury J.A., Storch G.A., Bohl D.L., Schuessler R.M., Torrence S.M., Lockwood M., Gaudreault-Keener M., Koch M.J., Miller B.W., Hardinger K.L. (2006). Prophylactic Versus Preemptive Oral Valganciclovir for the Management of Cytomegalovirus Infection in Adult Renal Transplant Recipients. Am. J. Transplant..

[22] Owers D.S., Webster A.C., Strippoli G.F.M., Kable K., Hodson E.M. (2013). Pre-Emptive Treatment for Cytomegalovirus Viraemia to Prevent Cytomegalovirus Disease in Solid Organ Transplant Recipients. Cochrane Database Syst. Rev..

[23] Singh N. (2001). Preemptive Therapy Versus Universal Prophylaxis with Ganciclovir for Cytomegalovirus in Solid Organ Transplant Recipients. Clin. Infect. Dis..

[24] Kliem V., Fricke L., Wollbrink T., Burg M., Radermacher J., Rohde F. (2008). Improvement in Long-Term Renal Graft Survival due to CMV Prophylaxis with Oral Ganciclovir: Results of a Randomized Clinical Trial. Am. J. Transplant..

[25] Reischig T., Jindra P., Hes O., Švecová M., Klaboch J., Třeška V. (2007). Valacyclovir Prophylaxis Versus Preemptive Valganciclovir Therapy to Prevent Cytomegalovirus Disease After Renal Transplantation. Am. J. Transplant..

[26] Witzke O., Hauser I.A., Bartels M., Wolf G., Wolters H., Nitschke M. (2012). Valganciclovir Prophylaxis Versus Preemptive Therapy in Cytomegalovirus-Positive Renal Allograft Recipients: 1-Year Results of a Randomized Clinical Trial. Transplantation.

[27] Manuel O., Kralidis G., Mueller N.J., Hirsch H.H., Garzoni C., van Delden C., Berger C., Boggian K., Cusini A., Koller M.T. (2013). Impact of Antiviral Preventive Strategies on the Incidence and Outcomes of Cytomegalovirus Disease in Solid Organ Transplant Recipients. Am. J. Transplant..

[28] Singh N., Winston D.J., Razonable R.R., Lyon G.M., Silveira F.P., Wagener M.M., Stevens-Ayers T., Edmison B., Boeckh M., Limaye A.P. (2020). Effect of Preemptive Therapy vs. Antiviral Prophylaxis on Cytomegalovirus Disease in Seronegative Liver Transplant Recipients with Seropositive Donors: A Randomized Clinical Trial. JAMA.

[29] Boehme K.W., Guerrero M., Compton T. (2006). Human Cytomegalovirus Envelope Glycoproteins B and H Are Necessary for TLR2 Activation in Permissive Cells. J. Immunol..

[30] Genini E., Percivalle E., Sarasini A., Revello M.G., Baldanti F., Gerna G. (2011). Serum Antibody Response to the gH/gL/pUL128–131 Five-Protein Complex of Human Cytomegalovirus (HCMV) in Primary and Reactivated HCMV Infections. J. Clin. Virol..

[31] Britt W.J., Vugler L., Butfiloski E.J., Stephens2 E.B. (1990). Cell Surface Expression of Human Cytomegalovirus (HCMV) Gp55-116 (GB): Use of HCMV-Recombinant Vaccinia Virus-Infected Cells in Analysis of the Human Neutralizing Antibody Response Downloaded From. http://jvi.asm.org/.

[32] Jonjić S., Pavić I., Polić B., Crnković I., Lucin P., Koszinowski U.H. (1994). Antibodies are not Essential for the Resolution of Primary Cytomegalovirus Infection but Limit Dissemination of Recurrent Virus. J. Exp. Med..

[33] Baraniak I., Kropff B., Ambrose L., McIntosh M., McLean G.R., Pichon S., Atkinson C., Milne R.S.B., Mach M., Griffiths P.D. (2018). Protection from Cytomegalovirus Viremia Following Glycoprotein B Vaccination is not Dependent on Neutralizing Antibodies. Proc. Natl. Acad. Sci. USA.

[34] Lilleri D., Zelini P., Fornara C., Zavaglio F., Rampino T., Perez L., Gabanti E., Gerna G. (2018). Human Cytomegalovirus (HCMV)-Specific T Cell but not Neutralizing or IgG Binding Antibody Responses to Glycoprotein Complexes gB, gHgLgO, and pUL128L Correlate with Protection Against high HCMV Viral Load Reactivation in Solid-Organ Transplant Recipients. J. Med. Virol..

[35] Boland G.J., Hené R.J., Ververs C., Haan M.A.M., Gast G.C. (2008). Factors Influencing the Occurrence of Active Cytomegalovirus (CMV) Infections after Organ Transplantation. Clin. Exp. Immunol..

[36] Chou S. (1989). Neutrallzing Antibody Responses to Reinfecting Strains of Cytomegalovirus in Transplant Recipients. J. Infect. Dis..

[37] Hughes D., Hafferty J., Fulton L., Friend P., Devaney A., Loke J., Welsh K.I., Handa A., Klenerman P. (2008). Donor and Recipient CMV Serostatus and Antigenemia after Renal Transplantation: An Analysis of 486 Patients. J. Clin. Virol..

[38] Humar A., Mazzulli T., Moussa G., Razonable R.R., Paya C.V., Pescovitz M.D., Covington E., Alecock E., Valganciclovir Solid Organ Transplant Study Group (2005). Clinical Utility of Cytomegalovirus (CMV) Serology Testing in High-risk CMV D+/R- Transplant Recipients. Am. J. Transplant..

[39] Reddehase M.J., Mutter W., Münch K., Bühring H.J., Koszinowski U.H. (1987). CD8-positive T Lymphocytes Specific for Murine Cytomegalovirus Immediate-Early Antigens Mediate Protective Immunity. J. Virol..

[40] Jonjić S., Pavić I., Lucin P., Rukavina D., Koszinowski U.H. (1990). Efficacious Control of Cytomegalovirus Infection after Long-Term Depletion of CD8+ T Lymphocytes. J. Virol..

[41] Jeitziner S.M., Walton S.M., Torti N., Oxenius A. (2013). Adoptive Transfer of Cytomegalovirus-Specific Effector CD4+T Cells Provides Antiviral Protection from Murine CMV Infection. Eur. J. Immunol..

[42] Reusser P., Riddell S., Meyers J., Greenberg P. (1991). Cytotoxic T-Lymphocyte Response to Cytomegalovirus after Human Allogeneic Bone Marrow Transplantation: Pattern of Recovery and Correlation with Cytomegalovirus Infection and Disease. Blood.

[43] Riddell S.R., Watanabe K., Goodrich J., Li C., Agha M., Greenberg P. (1992). Restoration of Viral Immunity in Immunodeficient Humans by the Adoptive Transfer of T cell Clones. Science.

[44] Walter E.A., Greenberg P.D., Gilbert M.J., Finch R.J., Watanabe K.S., Thomas E.D., Riddell S.R. (1995). Reconstitution of Cellular Immunity against Cytomegalovirus in Recipients of Allogeneic Bone Marrow by Transfer of T-Cell Clones from the Donor. N. Engl. J. Med..

[45] Reusser P., Attenhofer R., Thiel G., Cathomas G., Tamm M. (1999). Cytomegalovirus (CMV)–Specific T Cell Immunity after Renal Transplantation Mediates Protection from CMV Disease by Limiting the Systemic Virus Load. J. Infect. Dis..

[46] Bunde T., Kirchner A., Hoffmeister B., Habedank D., Hetzer R., Cherepnev G., Proesch S., Reinke P., Volk H.-D., Lehmkuhl H. (2005). Protection from Cytomegalovirus after Transplantation is Correlated with Immediate Early 1–Specific CD8 T Cells. J. Exp. Med..

[47] Sester M., Sester U., Gärtner B.C., Girndt M., Meyerhans A., Köhler H. (2002). Dominance of Virus-Specific CD8 T Cells in Human Primary Cytomegalovirus Infection. J. Am. Soc. Nephrol..

[48] Gamadia L.E., Rentenaar R.J., Baars P.A., Remmerswaal E.B.M., Surachno S., Weel J.F.L., Toebes M., Schumacher T.N.M., Berge I.J.M.T., van Lier R.A.W. (2001). Differentiation of Cytomegalovirus-Specific CD8+ T Cells in Healthy and Immunosuppressed Virus Carriers. Blood.

[49] Gerna G., Lilleri D., Chiesa A., Zelini P., Furione M., Comolli G., Pellegrini C., Sarchi E., Migotto C., Bonora M.R. (2011). Virologic and Immunologic Monitoring of Cytomegalovirus to Guide Preemptive Therapy in Solid-Organ Transplantation. Am. J. Transplant..

[50] Sester M., Sester U., Gartner B., Heine G., Girndt M., Mueller-Lantzsch N., Meyerhans A., Kohler H. (2001). Levels of Virus-Specific CD4 T Cells Correlate with Cytomegalovirus Control and Predict Virus-Induced Disease after Renal Transplantation1. Transplantation.

[51] Egli A., Binet I., Binggeli S., Jäger C., Dumoulin A., Schaub S., Steiger J., Sester U., Sester M., Hirsch H.H. (2008). Cytomegalovirus-Specific T-Cell Responses and Viral Replication in Kidney Transplant Recipients. J. Transl. Med..

[52] Nebbia G., Mattes F.M., Smith C., Hainsworth E., Kopycinski J., Burroughs A., Griffiths P.D., Klenerman P., Emery V.C. (2008). Polyfunctional Cytomegalovirus-Specific CD4+ and pp65 CD8+ T Cells Protect Against High-Level Replication After Liver Transplantation. Am. J. Transplant..

[53] Gamadia L.E., Remmerswaal E.B.M., Weel J.F., Bemelman F., van Lier R.A.W., Berge I.J.M.T. (2003). Primary Immune Responses to Human CMV: A Critical Role for IFN-γ–Producing CD4+ T Cells in Protection Against CMV Disease. Blood.

[54] Einsele H., Roosnek E., Rufer N., Sinzger C., Riegler S., Löffler J., Grigoleit U., Moris A., Rammensee H.-G., Kanz L. (2002). Infusion of Cytomegalovirus (CMV)–Specific T Cells for the Treatment of CMV Infection not Responding to Antiviral Chemotherapy. Blood.

[55] Crough T., Khanna R. (2009). Immunobiology of Human Cytomegalovirus: From Bench to Bedside. Clin. Microbiol. Rev..

[56] Walker S., Fazou C., Crough T., Holdsworth R., Kiely P., Veale M., Bell S., Gailbraith A., McNeil K., Jones S. (2007). Ex Vivo Monitoring of Human Cytomegalovirus-Specific CD8+ T-Cell Responses Using QuantiFERON^®^-CMV. Transpl. Infect. Dis..

[57] https://www.quantiferon.com/wp-content/uploads/2017/01/QuantiFERONCMVBrochure.pdf.

[58] Manuel O., Husain S., Kumar D., Zayas C., Mawhorter S., Levi M.E., Kalpoe J., Lisboa L., Ely L., Kaul D.R. (2013). Assessment of Cytomegalovirus-Specific Cell-Mediated Immunity for the Prediction of Cytomegalovirus Disease in High-Risk Solid-Organ Transplant Recipients: A Multicenter Cohort Study. Clin. Infect. Dis..

[59] Kumar D., Chernenko S., Moussa G., Cobos I., Manuel O., Preiksaitis J., Venkataraman S., Humar A. (2009). Cell-Mediated Immunity to Predict Cytomegalovirus Disease in High-Risk Solid Organ Transplant Recipients. Am. J. Transplant..

[60] Abate D., Saldan A., Mengoli C., Fiscon M., Silvestre C., Fallico L., Peracchi M., Furian L., Cusinato R., Bonfante L. (2013). Immunosorbent Spot and CMV Quantiferon Gamma Interferon-Releasing Assays in Assessing Risk of CMV Infection in Kidney. J Clin Microbiol..

[61] Cantisán S., Lara R., Montejo M., Redel J., Rodríguez-Benot A., Gutiérrez-Aroca J., González-Padilla M., Bueno L., Rivero A., Solana R. (2013). Pretransplant Interferon-γ Secretion by CMV-Specific CD8+ T Cells Informs the Risk of CMV Replication After Transplantation. Am. J. Transplant..

[62] Altaf M., Lineburg K., Crooks P., Rehan S., Matthews K.K., Neller M.A., Ambalathingal G.R., Sinha D., Grant M., Hopkins P.M.A. (2020). Pretransplant Cytomegalovirus-Specific Cellular Immunity and Risk of Viral Reactivation Following Lung Transplantation: A Prospective Cohort Study. J. Infect. Dis..

[63] Valle-Arroyo J., Aguado R., Páez-Vega A., Pérez A.B., González R., Fornés G., Torre-Cisneros J., Cantisán S. (2020). Lack of Cytomegalovirus (CMV)-Specific Cell-Mediated Immune Response Using QuantiFERON-CMV Assay in CMV-Seropositive Healthy Volunteers: Fact not Artifact. Sci. Rep..

[64] Lisowska K.A., Dębska-Ślizień A., Jasiulewicz A., Heleniak Z., Bryl E., Witkowski J.M. (2011). Hemodialysis Affects Phenotype and Proliferation of CD4-Positive T Lymphocytes. J. Clin. Immunol..

[65] Lisowska K.A., Radzka M., Witkowski J.M., Rutkowski B., Bryl E., Debska-Slizien A. (2010). Recombinant Human Erythropoietin Treatment of Chronic Renal Failure Patients Normalizes Altered Phenotype and Proliferation of CD4-positive T Lymphocytes. Artif. Organs.

[66] Albillos A., Lario M., Álvarez-Mon M. (2014). Cirrhosis-Associated Immune Dysfunction: Distinctive Features and Clinical Relevance. J. Hepatol..

[67] T-Track CMV—Cytomegalovirus In Vitro Diagnostic. https://www.lophius.com/products/t-track-cmv-elispot-kit.

[68] Kumar D., Chin-Hong P., Kayler L., Wojciechowski D., Limaye A.P., Gaber A.O., Ball S., Mehta A.K., Cooper M., Blanchard T. (2019). A Prospective Multicenter Observational Study of Cell-Mediated Immunity as a Predictor for Cytomegalovirus Infection in Kidney Transplant Recipients. Am. J. Transplant..

[69] Jarque M., Crespo E., Melilli E., Gutiérrez A., Moreso F., Guirado L., Revuelta I., Montero N., Torras J., Riera L. (2020). Cellular Immunity to Predict the Risk of Cytomegalovirus Infection in Kidney Transplantation: A Prospective, Interventional, Multicenter Clinical Trial. Clin. Infect. Dis..

[70] Abate D., Saldan A., Fiscon M., Cofano S., Paciolla A., Furian L., Ekser B., Biasolo M.A., Cusinato R., Mengoli C. (2010). Evaluation of Cytomegalovirus (CMV)–Specific T Cell Immune Reconstitution Revealed That Baseline Antiviral Immunity, Prophylaxis, or Preemptive Therapy but not Antithymocyte Globulin Treatment Contribute to CMV-Specific T Cell Reconstitution in Kidney Transplant Recipients. J. Infect. Dis..

[71] Sester U., Gartner B.C., Wilkens H., Schwaab B., Wossner R., Kindermann I., Girndt M., Meyerhans A., Mueller-Lantzsch N., Schafers H.-J. (2005). Differences in CMV-Specific T-Cell Levels and Long-Term Susceptibility to CMV Infection after Kidney, Heart and Lung Transplantation. Am. J. Transplant..

[72] San-Juan R., Navarro D., Garcia-Reyne A., Montejo M., Muñoz P., Carratala J., Len O., Fortun J., Munoz-Cobo B., Gimenez E. (2015). Effect of Long-Term Prophylaxis in the Development of Cytomegalovirus-Specific T-Cell Immunity in D+/R− Solid Organ Transplant Recipients. Transpl. Infect. Dis..

[73] Cantisán S., Páez-Vega A., Pérez-Romero P., Montejo M., Cordero E., Gracia-Ahufinger I., Martin-Gandul C., Maruri N., Aguado R., Solana R. (2016). Prevention Strategies Differentially Modulate the Impact of Cytomegalovirus Replication on CD8+ T-Cell Differentiation in High-Risk Solid Organ Transplant Patients. Antivir. Res..

[74] Andreani M., Albano L., Benzaken S., Cassuto E., Jeribi A., Caramella A., Giordanengo V., Bernard G., Esnault V., Seitz-Polski B. (2020). Monitoring of CMV-Specific Cell-Mediated Immunity in Kidney Transplant Recipients With a High Risk of CMV Disease (D+/R−): A Case Series. Transplant. Proc..

[75] Chiereghin A., Potena L., Borgese L., Gibertoni D., Squarzoni D., Turello G., Petrisli E., Piccirilli G., Gabrielli L., Grigioni F. (2018). Monitoring of Cytomegalovirus (CMV)-Specific Cell-Mediated Immunity in Heart Transplant Recipients: Clinical Utility of the QuantiFERON-CMV Assay for Management of Posttransplant CMV Infection. J. Clin. Microbiol..

[76] Westall G.P., Cristiano Y., Levvey B.J., Whitford H., Paraskeva M.A., Paul E., Peleg A.Y., Snell G.I. (2019). A Randomized Study of Quantiferon CMV-directed Versus Fixed-duration Valganciclovir Prophylaxis to Reduce Late CMV After Lung Transplantation. Transplantation.

[77] Poglajen G., Zemljič G., Frljak S., Cerar A., Andročec V., Božič T., Bitenc M., Okrajšek R., ŠEbeštjen M., Vrtovec B. (2020). QuantiFERON-CMV Guided Virostatic Prophylaxis after Heart Transplantation. J. Heart Lung Transplant..

[78] Kumar D., Mian M., Singer L., Humar A. (2017). An Interventional Study Using Cell-Mediated Immunity to Personalize Therapy for Cytomegalovirus Infection After Transplantation. Am. J. Transplant..

[79] Fernández-Ruiz M., Rodríguez-Goncer I., Parra P., Ruiz-Merlo T., Corbella L., López-Medrano F., Polanco N., González E., Juan R.S., Folgueira M.D. (2020). Monitoring of CMV-Specific Cell-Mediated Immunity with a Commercial ELISA-Based Interferon-γ Release Assay in Kidney Transplant Recipients Treated with Antithymocyte Globulin. Am. J. Transplant..

[80] Kim S.-H. (2020). Interferon-γ Release Assay for Cytomegalovirus (IGRA-CMV) for Risk Stratification of Posttransplant CMV Infection: Is It Time to Apply IGRA-CMV in Routine Clinical Practice?. Clin. Infect. Dis..

[81] Lúcia M., Crespo E., Melilli E., Cruzado J.M., Luque S., Llaudó I., Niubó J., Torras J., Fernandez N., Grinyó J.M. (2014). Preformed Frequencies of Cytomegalovirus (CMV)–Specific Memory T and B Cells Identify Protected CMV-Sensitized Individuals Among Seronegative Kidney Transplant Recipients. Clin. Infect. Dis..

[82] Schachtner T., Stein M., Reinke P. (2017). CMV-Specific T Cell Monitoring Offers Superior Risk Stratification of CMV-Seronegative Kidney Transplant Recipients of a CMV-Seropositive Donor. Transplantation.

[83] Chanouzas D., Small A., Borrows R., Ball S. (2018). Assessment of the T-SPOT.CMV Interferon-γ Release Assay in Renal Transplant Recipients: A Single Center Cohort Study. PLoS ONE.

[84] Jarque M., Melilli E., Bestard O., Crespo E., Manonelles A., Montero N., Torras J., Cruzado J.M., Luque S., Gil-Vernet S. (2018). CMV-specific Cell-mediated Immunity at 3-month Prophylaxis Withdrawal Discriminates D+/R+ Kidney Transplants at Risk of Late-onset CMV Infection Regardless the Type of Induction Therapy. Transplantation.

[85] Lee H., Park K.H., Ryu J.H., Choi A.-R., Hyun P.K., Lim J., Ha C.B., Kim S.I., Yang C.W., Chung B.H. (2017). Cytomegalovirus (CMV) Immune Monitoring with ELISPOT and QuantiFERON-CMV Assay in Seropositive Kidney Transplant Recipients. PLoS ONE.

[86] Donadeu L., Revilla-López E., Jarque M., Crespo E., Torija A., Bravo C., Arcos I.L., Meneghini M., Favà A., Román A. (2020). CMV-specific Cell-Mediated Immunity Predicts High level of CMV Replication after Prophylaxis withdrawal in Lung Transplant Recipients. J. Infect. Dis..

[87] Snyder L.D., Chan C., Kwon D., Yi J.S., Martissa J.A., Copeland C.A.F., Osborne R.J., Sparks S.D., Palmer S.M., Weinhold K.J. (2016). Polyfunctional T-Cell Signatures to Predict Protection from Cytomegalovirus after Lung Transplantation. Am. J. Respir. Crit. Care Med..

[88] Rogers R., Saharia K., Chandorkar A., Weiss Z.F., Vieira K., Koo S., Farmakiotis D. (2020). Correction to: Clinical Experience with a Novel Assay Measuring Cytomegalovirus (CMV)-Specific CD4+ and CD8+ T-Cell Immunity by Flow Cytometry and Intracellular Cytokine Staining to Predict Clinically Significant CMV Events. BMC Infect. Dis..

[89] Schoeppler K.E., Lyu D.M., Grazia T.J., Crossno J.T., Vandervest K.M., Zamora M.R. (2012). Late-Onset Cytomegalovirus (CMV) in Lung Transplant Recipients: Can CMV Serostatus Guide the Duration of Prophylaxis?. Am. J. Transplant..

[90] Jamal A.J., Husain S., Li Y., Famure O., Kim S.J. (2014). Risk Factors for Late-Onset Cytomegalovirus Infection or Disease in Kidney Transplant Recipients. Transplantation.

[91] Bestard O., Lucia M., Crespo E., van Liempt B., Palacio D., Melilli E., Torras J., Llaudó I., Cerezo G., Taco O. (2013). Pretransplant Immediately Early-1-Specific T Cell Responses Provide Protection for CMV Infection After Kidney Transplantation. Am. J. Transplant..

[92] Shin K.-H., Lee H.-J., Chang C.L., Kim E.J., Lim S., Lee S.J., Ryu J.H., Yang K., Choi B.H., Lee T.B. (2018). CMV Specific T Cell Immunity Predicts Early Viremia after Liver Transplantation. Transpl. Immunol..

[93] Paez-Vega A., Cantisan S., Vaquero J.M., Vidal E., Luque-Pineda A., Lobo-Acosta M.Á., Pérez A.B., Alonso-Moralejo R., Iturbe D., Monforte V. (2019). Efficacy and Safety of the Combination of Reduced Duration Prophylaxis followed by Immuno-Guided Prophylaxis to Prevent Cytomegalovirus Disease in Lung Transplant Recipients (CYTOCOR STUDY): An Open-Label, Randomised, Non-Inferiority Clinical Trial. BMJ Open.

[94] Gabanti E., Lilleri D., Scaramuzzi L., Zelini P., Rampino T., Gerna G. (2018). Comparison of the T-Cell Response to Human Cytomegalovirus (HCMV) as Detected by Cytokine Flow Cytometry and QuantiFERON-CMV Assay in HCMV-Seropositive Kidney Transplant Recipients. N. Microbiol..

[95] Åsberg A., Humar A., Jardine A.G., Rollag H., Pescovitz M.D., Mouas H., Bignamini A., Töz H., Dittmer I., Montejo M. (2009). Long-Term Outcomes of CMV Disease Treatment with Valganciclovir Versus IV Ganciclovir in Solid Organ Transplant Recipients. Am. J. Transplant..

[96] Turgeon N., Fishman J., Doran M., Basgoz N., Tolkoff-Rubin N., Cosimi A., Rubin R. (2000). Prevention Dof Recurrent Cytomegalovirus Disease in Renal and Liver Transplant Recipients: Effect of Oral Ganciclovir. Transpl. Infect. Dis..

[97] Humar A., Kumar D., Boivin G., Caliendo A.M. (2002). Cytomegalovirus (CMV) Virus Load Kinetics to Predict Recurrent Disease in Solid-Organ Transplant Patients with CMV Disease. J. Infect. Dis..

